# Catching a yawn: A multimodal experimental protocol for investigating sleepiness under visual and auditory yawning stimuli featuring human and digital characters

**DOI:** 10.1016/j.mex.2026.103921

**Published:** 2026-04-21

**Authors:** Zilu Liang, Kingkarn Khotchasing, Nhung Huyen Hoang

**Affiliations:** Ubiquitous and Personal Computing Lab, Kyoto University of Advanced Science (KUAS), Japan

**Keywords:** Contagious yawning, Sleepiness, Multimodal physiological sensing, Digital characters, Human-computer interaction, Arousal regulations

## Abstract

This protocol describes a laboratory-based experimental method for investigating whether exposure to contagious yawning stimuli is associated with measurable changes in subjective and physiological markers of sleepiness. The protocol employs a randomized within-subject crossover design comparing human versus digital character yawning stimuli, each presented with and without auditory cues. The method integrates validated subjective sleepiness assessments with multimodal physiological sensing including cardiovascular, electrodermal, pupillary, thermal, and neural activity measures. This protocol addresses a methodological gap in yawning research by shifting focus from yawning occurrence to downstream sleepiness-related state changes associated with yawning stimuli exposure, while also examining whether digitally presented yawning is comparable to human yawning.

Standardized experimental protocol for assessing sleepiness associated with contagious yawning stimuli exposure

Integration of subjective measures with multimodal physiological sensing for comprehensive state assessment

Direct comparison of yawning stimuli of human versus digital characters

## Graphical abstract

**Figure:**
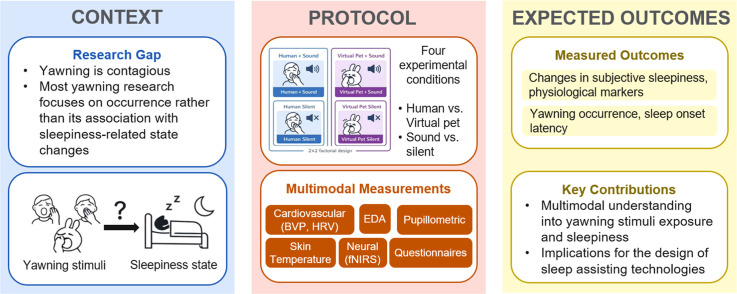

## Specifications table

**Table:** 

**Subject area**	Psychology
**More specific subject area**	Psychophysiology, contagious behavior, sleep health, human-computer interaction
**Name of your protocol**	Catch-a-yawn
**Reagents/tools**	▪ Smart wristband (EmbracePlus, Empatica Inc., US) ▪ Eye-tracking system (PupilCore, PupilLabs GmbH, Germany) ▪ Functional near-infrared spectroscopy system (Brite, Artinis Medical Systems, The Netherlands) ▪ Smartwatch (Google Pixel Watch, Google LLC, US) ▪ Video recording equipment ▪ Karolinska Sleepiness Scale (KSS) ▪ Munich Chronotype Questionnaire (MCTQ) ▪ Pittsburgh Sleep Quality Index (PSQI) ▪ Big Five Personality Test (BFPT)
**Experimental design**	Laboratory-based randomized within-subject crossover design with four experimental conditions: video of human yawning with sound, video of human yawning without sound, video of digital character yawning with sound, video of digital character yawning without sound. Each participant completes two sessions on separate days with randomized condition order.
**Trial registration**	Not applicable.
**Ethics**	This protocol was approved by the Ethics Review Board at Kyoto University of Advanced Science. Informed consent will be obtained from all participants prior to participation. All data will be anonymized and stored securely. Video recordings will be used only for behavioral coding and will be destroyed immediately after feature extraction.
**Value of the Protocol**	▪ Provides a systematic, replicable method for investigating sleepiness under contagious yawning stimuli using multimodal physiological assessment ▪ Addresses methodological gaps in yawning research by integrating subjective and objective sleepiness measures within a controlled experimental framework ▪ Establishes standardized procedures for comparing human versus digital character behavioral stimulus, which can inform the design of AI-driven sleep health technologies

## Background

Yawning is a ubiquitous human behavior that has been studied across both animal and human research contexts [1,2]. Beyond occurring spontaneously, yawning is socially contagious. Observing or hearing others yawn reliably increases the likelihood of yawning in the observer [[3], [4], [5]]. Contagious yawning has been linked to social cognition, empathy, and physiological regulation [3], and its neural mechanisms have been examined in both animals and humans [4,5]. Functional MRI research has implicated regions associated with social cognition and self-referential processing, including the medial prefrontal cortex, posterior cingulate cortex, and precuneus [[4], [5], [6]], as well as areas involved in attention and motor resonance. These findings suggest that contagious yawning engages networks related to both social processing and state regulation. This neurophysiological perspective motivates the inclusion of functional near-infrared spectroscopy (fNIRS) in the present study to assess prefrontal cortical activity during exposure to yawning stimuli.

Beyond its neural basis, a substantial body of research has also advanced our understanding of the physiological functions of yawning. In particular, the thermoregulatory or “brain cooling” hypothesis proposes that yawning serves to regulate brain temperature and maintain optimal neural functioning, with converging evidence from both animal and human studies [[7], [8], [9], [10]]. This perspective highlights the close relationship between yawning, arousal regulation, and physiological state changes.

Consistent with this view, experimental studies have examined how exposure to yawning stimuli influences behavior and cognition. For example, recent studies have demonstrated that viewing yawning stimuli can enhance vigilance and threat detection performance, such as improved detection of biologically relevant stimuli including snakes and spiders [[11], [12], [13]]. At the same time, yawning is commonly associated with fatigue, drowsiness, and transitions toward sleep [2,[14], [15], [16]], and prior studies have explored subjective sleepiness in relation to yawning behavior [2,14,15]. These findings suggest that yawning is closely linked to fluctuations in arousal, though its precise role in modulating internal states remains complex.

Yawning contagion is also multimodal. Although visual stimuli have been the most commonly studied, auditory contagious yawning has likewise been reported, with yawning sounds alone capable of eliciting yawning responses in listeners [17,18]. In addition, previous work has suggested that olfactory cues may contribute to yawning contagion, which further underscores the multimodal nature of this phenomenon [19]. These findings indicate that yawning can function as a socially transmitted cue across multiple sensory channels. In the present protocol, we focus specifically on visual and auditory yawning stimuli, while olfactory stimuli are not examined and are left for future investigation.

Despite these advances, comparatively fewer studies have systematically examined how exposure to yawning stimuli relates to integrated, multimodal changes in sleepiness and physiological arousal. Existing research has often focused on either behavioral outcomes (e.g., yawning occurrence or vigilance performance) or isolated measures of sleepiness, which limit the ability to characterize how yawning stimuli are associated with internal states across subjective and physiological domains simultaneously. A multimodal approach combining behavioral, subjective, and physiological measures may therefore provide a more comprehensive understanding of state changes related to exposure to contagious yawning stimuli.

At the same time, contemporary human–computer interaction increasingly involves digital agents, avatars, and animated representations of human behavior. Advances in AI and computer graphics have enabled these systems to display human-like expressions and social cues, and they are now widely deployed in digital health, education, customer support, and other interactive environments [20,21]. Such systems often rely on subtle behavioral signals to influence user engagement, affect, and cognitive state. Within this context, yawning represents a particularly intriguing but underexplored behavioral cue.

Previous studies have demonstrated that digitally presented yawning videos can elicit contagious yawning responses in humans [22,23], and even across species, such as in chimpanzees and orangutans exposed to digital or android yawning stimuli [24,25]. However, it remains unclear whether digitally presented yawning stimuli, particularly those generated by animal characters, are associated with sleepiness-related responses when assessed using multimodal psychophysiological measures.

Understanding whether exposure to yawning stimuli is associated with changes in sleepiness has important implications for both sleep research and the design of technologies intended to modulate arousal or relaxation [2]. Accordingly, this study seeks to advance this line of research by examining yawning videos featuring either humans or digital characters, presented with and without auditory cues, and assessing their associations with subjective and physiological indicators of sleepiness under controlled conditions. The protocol was developed to investigate four research questions: (1) whether exposure to human yawning videos is associated with sleepiness-related responses, (2) whether digital character yawning elicits comparable effects, (3) whether auditory yawning cues modulate these responses, and (4) how individual differences influence these associations.

## Description of protocol

### Overview

This protocol employs a laboratory-based, randomized within-subject crossover experimental design to examine the associations between contagious yawning stimuli exposure and subjective and physiological indicators of sleepiness. The protocol follows a 2 × 2 factorial structure manipulating: (1) stimulus type (human vs. digital character yawning) and (2) auditory content (with vs. without sound), yielding four experimental conditions.

Participants complete two sessions on separate days. In one session, they view human yawning videos under both auditory conditions in randomized order. In the other session, they view yawning videos of digital characters under the same auditory conditions. The order of sessions (human first vs. digital first) will be counterbalanced across participants. The order of conditions will be randomized and counterbalanced to minimize systematic order effects.

A within-subject design was selected to maximize sensitivity to condition-related differences while controlling for interindividual variability in baseline sleepiness, circadian preference, and autonomic functioning. To mitigate potential fatigue or habituation effects, experimental sessions will be conducted on separate days with a minimum interval of one-day between sessions.

The protocol is designed to examine relative differences across yawning stimulus conditions and within-subject changes over time, rather than to isolate yawning-specific causal effects relative to neutral stimulus exposure. Accordingly, non-yawning control conditions are not included. This design enables focused comparison across stimulus modalities and representations while maintaining a manageable experimental duration within a multimodal physiological recording context, which is consistent with prior studies using contagious yawning stimuli [26,27]. Pre-stimulus baseline recordings will be obtained prior to each condition, which allows within-subject quantification of changes relative to a stable resting reference.

**Fig. 1:**
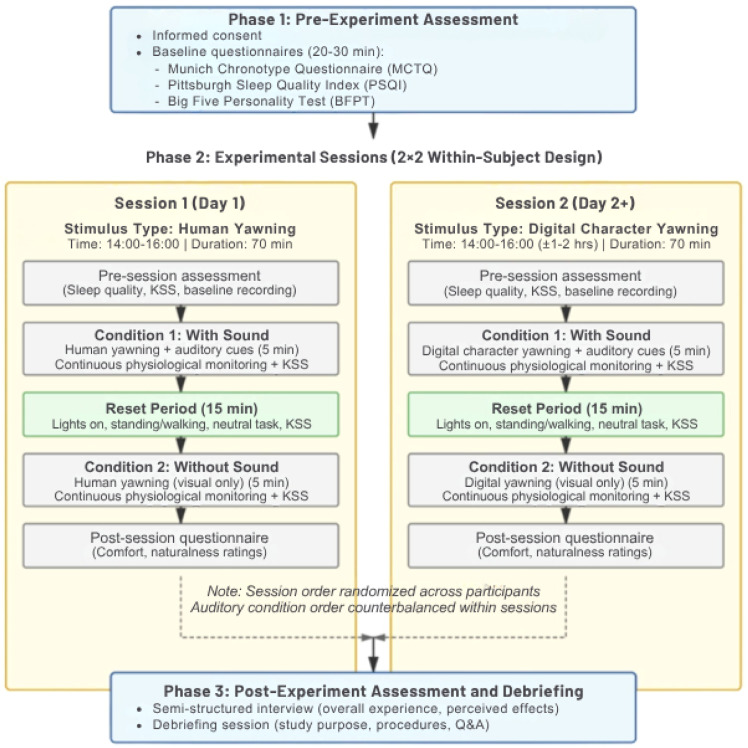
Protocol overview showing three-phase structure with pre-experiment assessment, two experimental sessions, and post-experiment assessment.

As illustrated in Fig. 1, the protocol consists of three sequential phases: (1) pre-experiment assessment, (2) two experimental sessions conducted on separate days, and (3) post-experiment assessment and debriefing. Each stimulus condition has a duration of approximately 5 min, resulting in a total stimulus exposure of approximately 10 min per session. This duration is selected to assess physiological state changes associated with yawning stimulus exposure while limiting fatigue and time-on-task effects, and is consistent with the 3–5 min range commonly employed in contagious yawning research [15,22].

### Participant selection and screening

Participants will be excluded if they meet any of the following criteria:

• Self-reported diagnosed mental health disorders
• Self-reported hearing impairments
• Inability to tolerate wearable sensing devices or experimental procedures
• Evidence of acute or chronic sleep deprivation, defined as insufficient sleep duration or extreme baseline sleepiness, identified during pre-session screening as described below.

To characterize sleep status and identify potential sleep deprivation prior to laboratory testing, a multi-tiered sleep assessment will be conducted. Wearable-based sleep monitoring will be conducted prior to the laboratory session. Participants who already use consumer smartwatches (e.g., Apple Watch, Fitbit, or Google Pixel Watch) will report their sleep timing data (bedtime and wake time) for the 3 nights preceding the application to participate in the study. For participants without a smartwatch, a Fitbit device will be provided to collect sleep timing and duration data for a minimum of 3 consecutive nights. Consumer-grade wearables have demonstrated reasonable accuracy, particularly for estimating total sleep duration, when compared with medical-grade devices [28,29]. In addition to wearable-based sleep tracking, baseline subjective sleepiness will be assessed before each laboratory session using the Karolinska Sleepiness Scale (KSS).

Participants meeting any of these criteria will be excluded to avoid potential confounding effects of acute or chronic sleep deprivation:

• Average sleep duration is <5 h across monitored nights as measured by a smartwatch
• Baseline KSS score ≥ 7

Sleep assessment will be conducted at the time of application, and only eligible participants will be notified and will subsequently be invited for pre-experiment assessment (see next section).

### Phase 1: pre-experiment assessment

Upon arrival, the participant will be provided with an overview of the study objectives and experimental procedures. They will be informed that the study examines responses to visual and audiovisual stimuli involving human and digital characters. To minimize expectation effects and participant demand characteristics, the specific focus on yawning stimuli will not be disclosed prior to stimulus presentation. The informed consent document will be reviewed in detail, and participants will be given the opportunity to ask questions before participation. Written informed consent will then be obtained prior to any data collection. Following consent, each participant will be assigned an anonymous identification code to ensure confidentiality throughout the study.

After the consent process, baseline questionnaire assessments will be administered to characterize individual sleep-related traits and personality factors. Circadian preference will be assessed using the Munich Chronotype Questionnaire (MCTQ), habitual sleep quality will be evaluated with the Pittsburgh Sleep Quality Index (PSQI), and personality traits will be measured using the Big Five Personality Test (BFPT). These measures will be collected to support descriptive characterization of the sample and to enable exploratory moderation analyses in subsequent data analysis.

Whenever possible, questionnaires will be provided in electronic format to facilitate efficient data entry and management. Participants will be instructed to complete the questionnaires at their own pace in a quiet and comfortable setting, with sufficient time allocated to minimize fatigue or response pressure.

### Phase 2: experimental sessions

The experimental phase consists of two intervention sessions conducted on separate days with a minimum one-day interval to minimize potential carryover effects related to fatigue or transient sleepiness. Each session corresponds to one stimulus type (human yawning vs. digital character yawning), with session order randomized across participants. Each session will follow an identical structure and last approximately 70 min. To minimize circadian confounding, experimental sessions will be scheduled at approximately the same time of day, typically between 14:00 and 16:00, consistent with prior findings that yawning exhibits circadian variation in both animals and humans [30,31]. The second session will be conducted within ±1–2 h of the first session whenever feasible. Sessions will be scheduled to avoid early morning or late evening when spontaneous yawning frequency is known to be naturally elevated [14,32,33].

#### Pre-session sleep quality and baseline sleepiness assessment

At the beginning of the session, participants will be greeted and their readiness to proceed will be confirmed. Sleep quality from the previous night, as measured by a smartwatch, will be reviewed. Baseline sleepiness will be assessed using the KSS. These data will be used to verify that the participant does not meet the exclusion criteria. Participants will then be instructed to observe the stimuli naturally without performing any explicit task. They will also be informed that they are free to yawn (or not) according to their natural inclination throughout the session, including during baseline recording.

#### Equipment and environment setup

The physiological sensing setup will begin with positioning the wrist-worn device. Prior to placement, the wristband battery will be confirmed to be fully charged. The device will be fitted on the participant’s non-dominant wrist and adjusted to achieve a snug yet comfortable fit that prevents sliding during the session.

Next, the eye-tracking system will be configured. Participants will be seated at a fixed distance of approximately 60–70 cm from a display screen while wearing the eye-tracking device, which is mounted on the participant’s head and positioned in front of the eyes. The fit and placement of the eye tracker will be adjusted to ensure comfort and optimal alignment with the participant’s eyes. A calibration sequence will then be performed in accordance with the manufacturer’s instructions [34]. Pupil detection quality will be checked immediately after calibration, and the experiment will proceed only after satisfactory tracking performance is confirmed.

The fNIRS system will then be configured. A Brite system (Artinis Medical Systems, The Netherlands) will be used, which operates at dual wavelengths (760 nm and 850 nm) with a sampling rate of 50 Hz. A cap with 27 channels (10 transimitters and 8 receivers; source-detector 3 cm apart) will be positioned on the participant’s head to cover bilateral prefrontal cortical regions. Optodes will be placed according to the international 10–20 system, with channels centered over positions approximately corresponding to the FpZ-F3-Cz-F4-FpZ regions. Prior to data collection, optode-scalp contact quality will be verified for each channel using the system’s built-in signal quality indicators [35]. Channels with consistently low signal quality indices will be flagged and excluded from analysis on a per-participant basis. In cases where contact quality is suboptimal, a shower cap will be fitted over the fNIRS cap to reduce ambient light interference and improve optode-scalp coupling. Data collection will proceed only after satisfactory signal quality is confirmed across a minimum of 20 channels. Raw optical signals will be converted to changes in oxygenated (HbO₂) and deoxygenated (HHb) hemoglobin concentration using the modified Beer-Lambert law as implemented in the NME-NIRS Python package [36].

Finally, a video camera will be positioned to capture the participant’s facial expressions throughout the session. The camera angle will be adjusted to ensure a clear view of the face, and ambient lighting will be optimized to support subsequent behavioral coding. A brief test recording will be conducted to confirm that facial features are clearly visible.

To ensure consistency across participants, the experimental environment will be standardized. Room temperature will be maintained between 20 and 22 °C throughout the session. Lighting conditions will be controlled at a moderate and consistent level. External noise and potential interruptions will be minimized as much as possible. Participants will be seated comfortably in a chair with back support, and the display screen will be positioned at eye level and at an appropriate viewing distance to reduce physical strain.

Following equipment setup, a five-minute baseline physiological recording will be conducted. This duration was selected based on prior studies examining the signal lengths required to reliably derive downstream physiological and cognitive indices [[37], [38], [39]]. Participants will be instructed to sit quietly in a reclining chair and relax while maintaining their gaze on a neutral fixation point displayed on the screen. All physiological signals will be recorded continuously during this period. To avoid introducing confounding influences, no conversation or additional instructions will be provided during the baseline recording.

#### Experimental procedure

Each experimental session includes two yawning stimulus conditions (with sound and without sound) presented in randomized, counterbalanced order. To minimize social presence effects on yawning responses [40,23], participants will remain alone in the experimental room throughout stimulus presentation, while the experimenter monitors the session remotely from an adjacent space via a live physiological data feed and video stream.

At the beginning of each condition, physiological recording will begin continuously, and participants will be instructed to watch the videos naturally and to maintain a comfortable and relaxed posture. No further instructions regarding yawning will be given at this stage, as participants will have been informed of their freedom to respond naturally at the pre-session assessment stage.

Stimulus presentation will then proceed for approximately 5 min. Each stimulus sequence will consist of approximately 50 discrete yawning events, each approximately 6 s in duration [41,42]. This duration is consistent with prior experimental yawning paradigms in contagious yawning research and falls within the commonly used 3–5 min exposure range [15,22]. During this period, all physiological signals will continue to be recorded continuously, and participant behavior will be monitored via the video recording. Particular attention will be given to signs of sleep onset such as sustained eye closure. If sleep onset occurs, the corresponding timestamp will be noted, and recording will continue without interruption.

Following stimulus presentation, a post-condition assessment period of approximately 10 min will be conducted. Video will be stopped, and subjective sleepiness will be assessed using the KSS. Participants will also complete a brief questionnaire regarding their yawning behavior, including whether they yawned or experienced an urge to yawn [40].

After the first condition, a standardized wakefulness reset period of approximately 15 min will be implemented. During this period, laboratory lighting will be increased, and participants will be encouraged to engage in light activities such as standing, brief walking, or casual movement. These activities are intended to promote wakefulness without inducing excessive physical exertion. Intentional or exaggerated upper-body stretching will not be encouraged, in order to minimize potential facilitation of yawning behaviour. Light conversation may be used when necessary to reduce accumulated sleep pressure. At the end of the reset period, subjective sleepiness will again be assessed using the KSS to confirm that participants have returned to an adequate level of alertness. If participants continue to exhibit signs of drowsiness, the reset period may be extended, or additional light activities may be introduced before proceeding.

The second condition will then follow the same procedure as described above. Upon the completion of both conditions (with and without sound), all physiological sensing equipment will be removed.

##### Post session questionnaires

At the end of the experimental session, participants will complete post-session questionnaires assessing the perceived comfort and naturalness of the yawning stimuli and provide open-ended feedback. If the completed session is the first session, the second session will be scheduled at this time.

### Phase 3: post-experimental assessment and debriefing

After completion of both experimental sessions, participants will take part in a final semi-structured interview to reflect on their overall experience, including perceived effects of the stimulus and any discomfort or fatigue experienced during the study.

Following the interview, a debriefing session will be conducted. During this session, the full purpose of the study (including the use of yawning stimuli) will be explained. Participants will be informed that the specific focus on yawning was not disclosed prior to the experiment in order to minimize expectation effects and demand characteristics. Participants will be given the opportunity to ask questions about the procedures or the potential findings. They will also be provided with the opportunity to withdraw data if desired.

### Yawning stimulus materials

Human yawning stimuli will be compiled from publicly available video recordings, drawing on sources such as YouTube (the “yawn-o-meter”), TikTok, and the YawnStim dataset [43]. Clips will be selected based on clear visibility of facial expressions and the presence of naturalistic yawning behavior. Each condition will include approximately 50 discrete, non-repeating yawning events, each lasting 6 s [41,42], resulting in a total stimulus duration of 5 min. The final stimulus set will include a balanced representation of male and female individuals.

Digital character yawning stimuli will be generated using generative AI tools to produce controlled yet diverse animated yawning clips. Stylized animal-like characters (e.g., cats and dogs) will be used to reduce potential confounds associated with human identity, attractiveness, or familiarity, while preserving clearly recognizable yawning behavior. Animal-like characters were selected because they are sufficiently human-dissimilar to avoid uncanny valley effects, yet sufficiently familiar to elicit socially contagious responses, consistent with prior evidence that contagious yawning occurs across species [44]. Each clip will be structured to reflect the typical temporal phases of a yawn, including gradual mouth opening, peak extension, and closure, with smooth motion transitions. Slight variations in timing and motion will be introduced to maintain naturalness while avoiding repetition. All generated clips will undergo manual selection and standardization to ensure experimental consistency. Clips with ambiguous expressions, unnatural motion, or technical artifacts will be excluded. Remaining clips will be standardized in duration, resolution, and presentation format.

For audiovisual conditions, yawning sounds will be synchronized with the peak mouth-opening phase of each clip, while silent conditions will use identical clips without audio. For the human yawning clips with audio, the original audio from the source clips will be retained. For digital pet clips and human yawning clips without audio, yawning sounds will be synthesized using audio generation tools. Generated sounds will be standardized in amplitude and duration and evaluated for perceived naturalness prior to use. Stimulus sequences will be matched across human and digital pet conditions in terms of the number of yawns, inter-yawn intervals, and total duration.

All candidate clips (both human and digital pet) will be independently evaluated by two trained research assistants who are blind to the study hypotheses. Each clip will be assessed based on the clarity of yawning behavior, absence of ambiguity with other facial actions, and technical quality. Only clips with unanimous agreement across all criteria will be included. Inter-rater agreement will be quantified using Cohen’s *K*, and any disagreements will be resolved through adjudication by a third rater.

### Data collection and management

#### Data collection overview

Physiological data will be collected continuously during the experimental sessions using a multimodal sensing setup. Questionnaire data will be collected at multiple timepoints. Table 1 summarizes all data that will be collected and their corresponding instruments.

**Table 1:** Data collection summary.

Instruments	Data collected	Data collection timing
Consumer-grade smartwatches	Sleep duration, sleep schedule	Participants screening, and throughout the experiment days
KSS questionnaire	Baseline and post-condition subjective sleepiness levels	Participants screening, pre-experiment session baseline, post-condition, end of wakefulness reset period
MCTQ questionnaire	Chronotype	Pre-experiment assessment
PSQI questionnaire	Subjective sleep quality over the past one month	Pre-experiment assessment
Big Five Personality	Personality traits	Pre-experiment assessment
Research-grade smart wristband	Blood volume pulse (BVP), heart rate variability (HRV), electrodermal activity (EDA), 3-axis acceleration, and skin temperature	Experimental sessions
Eye-tracking system	Pupil diameter of both eyes	Experimental sessions
Functional near-infrared spectroscopy (fNIRS) system	Changes in HbO_2_ and HHb in the prefrontal cortical regions, head movements captured by 3-axis acceleration	Experimental sessions
Video recorder	Used solely to identify observable yawning behavior (counts of yawning, eye closure duration); recordings will be permanently deleted after feature extraction	Experimental sessions
Simple Likert Scales	Stimulus naturalness and comfort ratings	Experimental sessions
Semi-structured interview	Post-experiment interview responses	Post-experimental assessment

#### Data storage and security

All data collected in this study will be handled in accordance with strict data protection and ethical guidelines. To protect participant privacy, data anonymization will be implemented at the time of enrollment. Each participant will be assigned a unique anonymous code, which will be used to label all data files. All physiological data files will be kept on a password-protected offline computer, while questionnaire data will be stored in encrypted digital formats. Video recordings will be stored only temporarily on a secure offline device for the sole purpose of behavioral feature extraction, such as identifying yawning occurrences and sleep onset timing. Once these behavioral codes have been extracted, all video recordings will be permanently deleted. Only the derived behavioral codes will be retained, and these will be incorporated into a fully de-identified dataset for analysis. Consistent file naming conventions (e.g., “ID_SessionNumber_Condition_DataType_Date”) will be used throughout the study to facilitate data processing, improve traceability, and reduce the likelihood of handling errors.

#### Data pre-processing

Physiological and behavioral data will be time-aligned across sensing modalities using synchronized timestamps. For each yawning condition, physiological signals will be segmented into predefined analysis windows relative to stimulus onset.

Artifact removal will then be performed separately for each sensing modality. For wristband-based signals, visual inspection and accelerometer data will be used to identify motion artifacts, signal dropout, and periods of excessive movement, which will be excluded from further analysis. For eye-tracking data, periods of sustained eye closure indicative of potential sleep onset as well as poor-quality pupil measurements, will be excluded. For fNIRS data, standard preprocessing procedures including bandpass filtering and motion artifact correction will be applied, followed by the removal of segments affected by low signal quality. Raw optical signals will subsequently be converted into HbO_2_ and HHb concentration changes.

To ensure that analyses capture stimulus-related physiological responses rather than transitions into sleep, only data recorded prior to identified sleep onset will be included in confirmatory analyses. Data segments affected by excessive artifacts, signal loss, or sustained eye closure will be excluded from primary analyses. Baseline correction will be applied to all physiological measures to account for inter-individual differences in resting physiological states. Baseline values will be calculated for each participant using pre-stimulus periods and subtracted from condition-specific values or expressed as percentage changes, as appropriate.

Following signal cleaning, physiological features will be extracted for each experimental condition. BVP will be used to compute mean heart rate and HRV metrics, including SDNN, RMSSD, and frequency-domain measures. EDA data will be analyzed to derive tonic skin conductance level and phasic skin conductance responses. Pupil data will be used to calculate mean pupil diameter, pupil diameter variability and pupillary unrest index (PUI). Skin temperature data will be summarized using mean peripheral temperature and changes relative to baseline. For fNIRS data, temporal patterns of cortical oxygenation changes will be extracted. Finally, descriptive statistics will be used to summarize participant characteristics, baseline sleep measures, and data completeness.

#### Data analysis

The protocol is designed to examine relative differences across yawning stimulus conditions and within-subject changes over time, rather than to establish yawning-specific causal effects relative to neutral stimulus exposure. As such, findings will be interpreted in terms of associations with yawning stimulus exposure and relative differences across conditions, rather than as evidence of yawning-specific causal effects.

The primary outcome will be the change in subjective sleepiness, as assessed using post-condition KSS ratings. Analyses will focus on within-subject changes in KSS across experimental conditions, as well as changes relative to baseline measurements.

Secondary outcomes will include physiological markers associated with sleepiness and arousal, including autonomic nervous system activity (e.g., HRV, EDA), skin temperature variation, pupil size dynamics, and neural activity patterns associated with reduced alertness. These measures are selected based on prior literature demonstrating their sensitivity to changes in vigilance and alertness. For example, HRV has been shown to increase with sleepiness and reduced vigilance, often reflecting shifts toward parasympathetic dominance, particularly in low-frequency bands [45,46]. Similarly, pupillary measures such as the PUI have also been associated with increased sleepiness and reduced alertness [47,48]. In addition, decreases in oxygenated and total hemoglobin in prefrontal regions, as measured by fNIRS, have been linked to fatigue and reduced alertness [49].

While these physiological measures have been widely used as indices of arousal and vigilance, they are not universally established as direct measures of subjective sleepiness. Accordingly, these measures will be interpreted as complementary indicators of arousal-related processes in conjunction with subjective and behavioral measures. Analytical focus will therefore be placed on changes in physiological signals relative to baseline and on their associations with subjective sleepiness, rather than attempting to derive a single direct physiological measure of sleepiness.

Exploratory outcomes will include behavioral indicators of sleepiness, such as yawning occurrence and instances of sleep onset observed during the experiment. Additional exploratory analyses will examine the relationship between yawning behavior and both subjective and physiological outcomes. These relationships will be modeled using mixed-effect approaches where appropriate, which allows for the inclusion of both within-subjects (e.g., condition, time) and between-subjects (e.g., chronotype, baseline sleep quality) factors.

Statistical significance will be evaluated using two-sided tests with an alpha level of 0.05 unless otherwise specified. Results will be reported using effect sizes (e.g., estimated mean differences as quantified by Cohen’s *d* or Hedge’s *g*) alongside 95% confidence intervals and p-values. Given the exploratory nature of several outcomes, findings will be interpreted with appropriate caution, and emphasis will be placed on the direction, magnitude, and consistency of observed effects rather than statistical significance alone. The data analysis plan is summarized in Table 2.

**Table 2:** Data analysis plan.

Analysis Level	Procedure
Primary outcome analysis	▪ Analyze changes in KSS using linear mixed-effects models to account for the within-subject repeated-measures design ▪ Fixed effects will include stimulus type (human vs digital character yawning), auditory condition (with sound vs without sound), and their interaction. ▪ Subject will be included as a random effect with a random intercept to account for interindividual differences in baseline sleepiness. ▪ Where model convergence permits, random slopes for condition effects may be explored. ▪ Conduct planned comparison [50] to examine differences in subjective sleepiness between human and digital yawning stimuli, differences between auditory conditions (with vs without sound), and the interaction between stimulus type and auditory condition.
Secondary outcome analysis	▪ Apply the mixed-effects modeling framework used for primary outcome analysis to each physiological outcome, including HRV, EDA, skin temperature, pupil dynamics, and fNIRS measures. ▪ Analyses will focus on changes relative to baseline and associations with experimental conditions. ▪ Where appropriate, multiple comparison adjustments (e.g., Bonferroni or false discovery rate correction) will be reported as supplementary information.
Exploratory analysis	▪ Examine sleep onset occurrence and sleep onset latency based on behavioral and physiological indicators. ▪ Count observable yawning events by condition. ▪ Examine the relationship between yawning frequency and outcome measures (KSS scores and physiological measures) using mixed-effect models. ▪ Where temporal resolution permits, conduct event-based analyses to examine short-term physiological responses surrounding yawning events. ▪ Explore moderation effects by including interaction terms in mixed-effects models (e.g., chronotype, habitual sleep quality, baseline sleepiness, and personality traits).
Model diagnostics	▪ Inspect Q-Q plots of residuals to assess normality. ▪ Plot residuals vs fitted values to check homoscedasticity. ▪ Use formal diagnostic tests where appropriate (Shapiro-Wilk, Breusch-Pagan). ▪ Report any violations and their potential impact on inference and apply transformations if necessary.

### Limitations

Several limitations should be acknowledged. First, the laboratory-based setting may limit ecological validity relative to real-world contexts in which yawning and sleepiness naturally occur. Second, the protocol does not include a non-yawning control condition. Although baseline and post-condition measurements allow for within-subject assessment of state changes, the absence of a neutral comparison condition limits the ability to attribute observed changes uniquely to yawning stimuli, as alternative explanations such as time-on-task effects, fatigue, or repeated exposure cannot be fully excluded. Third, while behavioral indicators of sleep onset and physiological correlates of reduced arousal will be assessed, the study does not include polysomnography. Therefore, precise sleep onset latency cannot be determined. Despite these limitations, this protocol establishes a methodological framework for investigating how human-like behavior presented by digital agents is associated with subjective and physiological states, which provides a foundation for future confirmatory studies and real-world deployments.

Findings from executing this protocol will have several important implications. From a theoretical perspective, it contributes to a more functional understanding of whether exposure to contagious yawning stimuli is associated with changes in sleepiness and arousal regulation, rather than focusing solely on yawning frequency. By integrating subjective, physiological, and behavioral indicators, the study informs ongoing debates regarding the potential role of yawning stimuli exposure in sleep-related state modulation. From an applied perspective, the findings may inform the design of non-pharmacological approaches for modulating arousal and relaxation. In particular, evidence that digitally presented yawning stimuli are associated with sleepiness-related responses would have implications for the design of AI-driven avatars, digital companions, and interactive health technologies, where subtle behavioral cues may be leveraged to influence user state without explicit instruction.

Importantly, even in the absence of strong effects, the study will help delineate the conditions under which exposure to contagious yawning stimuli is not meaningfully associated with sleepiness-related states. Such null or weak findings would provide valuable insight for refining theoretical models and informing the design of future digital interventions.

## Related research article

None*.*

## CRediT author statement

**Zilu Liang**: Conceptualization, Methodology, Visualization, Resources, Supervision, Writing - Original draft preparation, Writing - Reviewing and Editing. **Kingkarn Khotchasing**: Conceptualization, Methodology. **Nhung Huyen Hoang**: Conceptualization, Writing-Reviewing and Editing.

## Declaration of competing interest

The authors declare that they have no known competing financial interests or personal relationships that could have appeared to influence the work reported in this paper.

## Data Availability

No data was used for the research described in the article.
